# Editorial: Spanish psycholinguistics in the 21st century

**DOI:** 10.3389/fpsyg.2022.964497

**Published:** 2022-08-04

**Authors:** Jon Andoni Duñabeitia, José Antonio Hinojosa, Claudia Poch, Alice Foucart

**Affiliations:** ^1^Centro de Investigación Nebrija en Cognición (CINC), Universidad Nebrija, Madrid, Spain; ^2^UiT the Arctic University of Norway, Tromsø, Norway; ^3^Instituto Pluridisciplinar, Universidad Complutense de Madrid, Madrid, Spain; ^4^Facultad de Psicología, Universidad Complutense de Madrid, Madrid, Spain

**Keywords:** Spanish, psycholinguistics, cognitive science, language, language science

The year 2022 marks the 500th anniversary of the death of Antonio de Nebrija, the first Hispanic humanist. With scientific rigor as the standard of his activity, Nebrija not only wrote the first grammar of Spanish (thus being the first written grammar of a modern European language), but also worked as a translator, lexicographer, linguist, and historian, among others. In the essence of Antonio de Nebrija, a humanist concerned with the scientific study of grammar, lexicon, and orthography, we surely find one of the foundational bases of a series of scientific areas that, years later, have resulted in what we now know as psycholinguistics and applied linguistics.

Using the example of the multiplicity of interests of Antonio de Nebrija as a humanist scientist devoted to the study of Spanish more than 500 years ago, today we can see how research on the second most widely spoken language in the world, used by nearly 500 million people, is in exceptional health. More and more laboratories are flourishing inside and outside Spanish-speaking countries that dedicate their research work to generating knowledge about language processing and production, using their vernacular language as a spearhead. Moreover, in recent years we have observed how many international research centers located in countries where Spanish is not one of the official languages have also oriented part of their scientific activity to the study of Spanish. Hence, it is not difficult to find centers specialized in the psycholinguistic or neurolinguistic study of people who speak Spanish as their first language, just as it is not difficult to find laboratories that explore the learning and processing of Spanish as a second language, additional language, heritage language or foreign language.

Whether for its processing or learning as a first language or as an additional language, Spanish constitutes in itself a wealth of particularities of great socio-linguistic relevance that makes it an incessant source of research questions. Both because of the constitution of its lexicon influenced by the Romans, Arabs, Celts, and other cultures, and because of the large number of dialectal varieties that expand in different continents, Spanish is a privileged language that allows psycholinguistic approaches to socio-linguistic aspects questions. Moreover, its prototypical subject-verb-object syntactic structure and the possibility of pro-dropping of the subject, together with the inflectional complexity of the language that requires high agreement demands, places Spanish in an advantageous position to explore linguistic processes that would be difficult (if not impossible) to investigate in different languages.

This being the case, it is not surprising that the beginning of the twenty-first century has represented a clear transition toward the professionalization of experimental research in Spanish psycholinguistics and neurolinguistics with the aim of shedding light on the cognitive processes that underlie the acquisition, learning, perception, or production of language. The growing interest in the use of Spanish as a tool for cracking the code of the linguistic macrosystem or of some of the associated processes, as well as the progressive appearance and consolidation of research centers and laboratories in social contexts where Spanish plays a relevant role (whether as a majority language or not), has been reflected in the increase in the number of related scientific publications. By way of illustration, a search carried out in June 2022 in a commonly used scientific portal such as Scopus, using “Spanish,” “language,” and “cognition” as search terms in the title, keywords, and abstract of the available sources, shows that from 2000 to 2020 the increase in publications was an impressive 900%. Of the total number of publications found, slightly more than half correspond to the areas of Psychology (21.4%), Medicine (22.4%), and Neuroscience (11.5%). Importantly, in order to understand the transdisciplinary nature of the work carried out in Spanish Psycholinguistics, it is important to highlight that the areas of Social Sciences (15.8%) and Arts and Humanities (15.6%) also account for a very significant portion of the scientific activity carried out. After the XV International Symposium of Psycholinguistics held in Madrid in June 2021 and aligned with the multiple interests of dozens of research groups around the world (as was also the case with the multiple interests of Antonio de Nebrija), this Research Topic offers an overview of the state of the main lines of experimental research on Spanish psycholinguistics.

The XV International Symposium of Psycholinguistics had more than 200 attendees and nearly 150 different participating institutions, and with 72 poster presentations and 79 oral communications, in addition to the keynote lectures, it demonstrated that scientific research in psycholinguistics, neurolinguistics, and applied linguistics on Spanish or in Spanish is at a moment of splendor, and the collection of articles that we present here give a good account of this.

Using various methodologies such as eye-tracking and electrophysiology, recent research in psycholinguistics has employed Spanish as the language to better understand linguistic, cognitive, and societal concepts in native and bilingual contexts. This Research Topic offers a case of the paradigmatic example of an overview of this work.

With the focus on word recognition processes, Marcet et al. examined whether the slower word processing times recently observed when accent marks were omitted [e.g., *carcel* derived from *cárcel* (prison)] was due to the experimental designs used or to the fact that accent-marked vowels are represented by the same orthographic units during word recognition and reading. They concluded that the effect is task-dependent, suggesting that the omission of accent marks may not generate a reading cost. Word recognition processes were also put to the test, in this case in bilingual contexts, in the article by Comesaña et al. They investigated whether the flexible letter position coding observed during native word recognition (e.g., *cholocate* misread as chocolate; see Perea et al., 2008) occurs similarly during bilingual word recognition. Their results revealed differences depending on the language cue and have implications for the models of bilingual word recognition. Regarding syntactic processing, Baron et al. examined grammatical gender processing in school-age Spanish-English bilingual children using a visual-world paradigm. They observed an asymmetry in the usage of gendered articles that was modulated by the frequency of use of the bilinguals' two languages. Finally, in relation to second language processing, Margaza and Gavarró studied the expression and position of subjects in Greek speakers of Spanish, and they report results that go against the predictions of different versions of the Interface Hypothesis (e.g., Sorace and Filiaci, 2006).

A different series of articles in this Research Topic focus on emotions and emotional language processing, illustrating a great deal of attention put on this topic by Spanish psycholinguists (see Hinojosa et al., 2020). In their article, Veitez et al. tried to unravel the mystery about the negative valence bias by evaluating the contribution of arousal to unpleasant word recognition. Their event-related brain potential (ERP) data obtained in a lexical decision task revealed the mediating role played by arousal in the emergence of the negative valence effects in word recognition. In a study exploring oscillatory activity, Santaniello et al. examined the impact of approach and avoidance motivational systems in the processing of emotional words. To do so, they compared frontal alpha asymmetries and brain oscillations triggered by anger and fear words. Their results suggested that motivational features play a role in the representation and processing of emotional words. Finally, Hatzidaki and Santesteban presented data from another ERP study showing that number agreement is sensitive to the affective nature of semantic information. Interestingly, their data clarified the different stages of language processing at which emotional information may impact syntactic parsing.

Lastly, two of the articles presented in the current Research Topic focused on the societal changes that could impact language processing. The research article by Pilgun et al. explored the perception of the COVID-19 pandemic by users of Spanish, German, and Russian. The analysis of large databases built from various social sources using a neural network approach revealed similarities and differences across the speakers of the languages in relation to various aspects such as attitudes toward vaccination. Finally, in their article, Planelles Almeida et al. compiled a dataset of oral interactions in Spanish by migrants and refugees from underrepresented countries and different language backgrounds. Their dataset represents an important tool for researchers in psycholinguistics who study L2 spoken language comprehension and processing.

As we can see, this Research Topic is a good example of the variety of methodological and theoretical approaches to the study of language in the Spanish psycholinguistic field. From compilations of oral productions in interactions with non-native speakers of the language to analyses of brain potentials or neural oscillations to explore the interface between language and emotion, and to studies on orthographic processing, this collection of articles shows the good scientific health that this field currently enjoys, and the solid commitment that is being made to the internationalization of results from dozens of research teams working in areas related to the cognitive science of language. Due to its history and development, and due to the relevance that the research groups focusing on Spanish Psycholinguistics have gained internationally, we are certain that the different lines of work of these laboratories will continue to allow us to address translinguistic questions of high scientific significance. Moreover, our analysis of the current situation of the specific area of Spanish Psycholinguistics makes us believe that we are already on a journey directed toward understanding the reality of overcoming the barriers of the WEIRD societies (Western, Educated, Industrialized, Rich, and Democratic; see Henrich et al., 2010). In a scientific world in which generalized Anglocentrism continues to prevail, the progressive advance of the work carried out in the field of Spanish Psycholinguistics can help break down knowledge barriers, achieving higher rates of representativeness, especially if we consider the sociolinguistic richness of Spanish.

## Author contributions

All authors listed have made a substantial, direct, and intellectual contribution to the work and approved it for publication.

## Conflict of interest

The authors declare that the research was conducted in the absence of any commercial or financial relationships that could be construed as a potential conflict of interest.

## Publisher's note

All claims expressed in this article are solely those of the authors and do not necessarily represent those of their affiliated organizations, or those of the publisher, the editors and the reviewers. Any product that may be evaluated in this article, or claim that may be made by its manufacturer, is not guaranteed or endorsed by the publisher.
